# Remdesivir resistance in transplant recipients with persistent COVID-19

**DOI:** 10.21203/rs.3.rs-1800050/v1

**Published:** 2022-06-29

**Authors:** John I Hogan, Ralf Duerr, Dacia Dimartino, Christian Marier, Sarah Hochman, Sapna Mehta, Guiqing Wang, Adriana Heguy

**Affiliations:** NYU Long Island School of Medicine; NYU Grossman School of Medicine; NYU Langone Health; NYU Langone Health; NYU Grossman School of Medicine; NYU Grossman School of Medicine; NYU Grossman School of Medicine; NYU Grossman School of Medicine

**Keywords:** remdesivir, SARS-CoV-2, resistance, mutation, immunesuppression, transplant

## Abstract

The medical community currently lacks robust data regarding the incidence, prevalence, and clinical significance of mutations associated with resistance to severe acute respiratory syndrome coronavirus-2 (SARS-CoV-2) therapeutics. This report describes two renal transplant recipients who, after remdesivir exposure, developed a *de novo* V792I RNA-dependent RNA polymerase (RdRp) mutation that has recently been found to confer resistance to remdesivir *in vitro*. To the best of our knowledge, this publication is the first to document the emergence of V792I in patients treated with remdesivir. Our work underscores the critical need for augmented efforts to identify concerning mutations and address their clinical implications.

## Introduction

While measures to curb the spread of SARS-CoV-2, a pathogen that has claimed the lives of more than a million Americans, continue to ease in many parts of the United States, the risk of adverse outcomes related to infection in immunocompromised hosts remains substantial^1,2^. In a recent meta-analysis that included nearly 12,000 solid organ transplant (SOT) recipients, vaccination against COVID-19 elicited a humoral response that was significantly reduced compared to that of immunocompetent hosts^3^. Though vaccination results in a markedly decreased risk of severe infection in the immunocompetent majority, a diminished response to vaccination translates to a greater risk of severe disease and death in SOT recipients^4^.

Given the substantial risk associated with SARS-CoV-2 infection in SOT recipients, proactive therapy is warranted in patients requiring hospitalization. Remdesivir, a prodrug of the nucleoside analog GS-441524 that inhibits the activity of RdRps in multiple RNA viruses, represents the first direct-acting antiviral approved by the FDA for the treatment of COVID-19^5^. Very recently, a case of *de novo* remdesivir resistance was described in a patient who experienced a protracted course of COVID-19 while receiving rituximab and bendamustine for lymphoma^6^. The risk of developing resistance to COVID-19 therapeutics during the course of treatment and the precise prevalence of clinically significant mutations in the community are not known.

## Case 1

A patient in their 60s with a history of hypertension, diabetes, and vascular disease underwent deceased donor kidney transplant (DDKT) addressing end-stage renal disease (ESRD). Prior to transplant, the patient received two doses of the BNT162b2 vaccine. Initially treated with induction immunosuppression including basiliximab and methylprednisolone, an elevated anti-A titer in the blood type B recipient prompted a transition from basiliximab to anti-thymocyte globulin (ATG). Cytopenias and delayed graft function (DGF) requiring hemodialysis increased suspicion for calcineurin inhibitor (CNI) toxicity, tacrolimus was discontinued, and the patient received an augmented corticosteroid regimen. Ongoing DGF and cytopenias prompted a biopsy of the graft on postoperative day seven, which suggested acute cellular rejection and antibody-mediated rejection. The patient was treated with another dose of ATG, a course of plasmapheresis, and was ultimately transitioned to maintenance immunosuppression including prednisone, mycophenolate, and belatacept. The early postoperative course was further complicated by donor-derived hepatitis C virus (HCV) infection, an intra-abdominal urine leak requiring drainage and nephrostomy tube placement, and pyelonephritis.

Six months after transplant, the patient developed the new onset of malaise, fatigue, cough, and fever. On admission, nasopharyngeal RT-PCR was positive for SARS-CoV-2 with a cycle threshold (Ct) of 27.1. Genomic sequencing identified the B.1.529 (Omicron) subvariant BA.1.1. Not requiring oxygen at that time, the patient received a five-day course of remdesivir, experienced improvement in symptomatology including defervescence, and was discharged.

**Figure 1:**
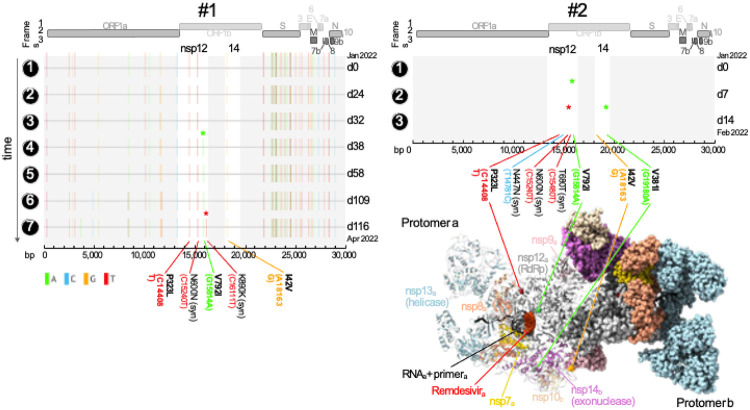
Independent acquisition of the remdesivir resistance mutation nsp-V792I in two immunocompromised patients. Full genome mutation profiles of SARS-CoV-2 viruses in longitudinal specimens of two immunocompromised patients treated with remdesivir. Basepair (bp) mutations compared to the Wuhan- Hu-1 reference are shown as ticks, color-coded according to the legend on the lower left. Bp and corresponding amino acid (aa) mutations in nsp12 (RNA-dependent RNA polymerase [RdRp]) and nsp14 (containing 3'-to-5' exoribonuclease proofreading activity) are labeled and shown in bold if non-synonymous. The longitudinal acquisition of mutations in nsp12 and 14 are highlighted by colored asterisks. Full genome maps are shown on top. The timeline is shown on the y-axis where time points are indicated on the left and days (d) elapsed since the first COVID-19 sampling per patient on the right of each plot. A 3D protein structure of the multi-domain polymerase complex is shown in its active dimeric form. Each domain is colored differently and labeled in the protomer that is shown in ribbon representation, whereas the other domains are shown in sphere representation, respectively. Nsp14 (exonuclease activity) and its co-factor nsp10 convey RNA proofreading in trans and are thus highlighted/shown as ribbons in protomer b together with the other domains in protomer a. The non-synonymous mutations in nsp12 and 14 as well as remdesivir are highlighted and labeled. The structure of the polymerase complex dimer is based on pdb 7egq with remdesivir added by structural overlay of a remdesivir-bound nsp12 complex (pdb 7l1f)^21,22^.

**Figure 2:**
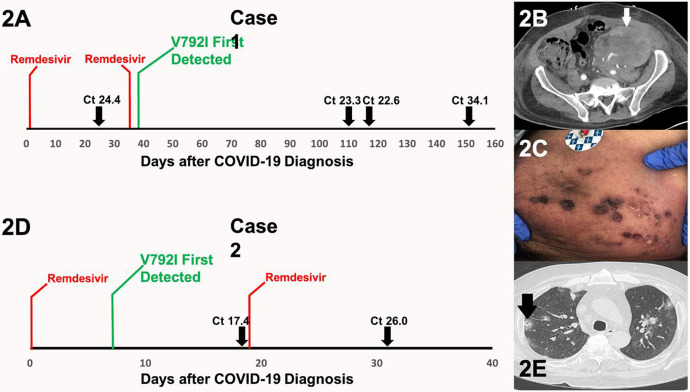
**2A** Relation between the timing of remdesivir exposure and the subsequent development of the *de novo* RdRp V792I mutation in Case 1. Select Ct values are provided at points when the patient was symptomatic. A Ct of 34.1 was obtained 153 days after the diagnosis of COVID-19 when the patient experienced durable resolution of all symptoms associated with SARS-CoV-2 infection. **2B** Case 1 CT of the abdomen demonstrating mass-like thickening along the renal graft (arrow) contiguous with the abdominal wall. **2C** Case 1 cutaneous findings along the left abdomen overlying the renal graft. Biopsy yielded cells consistent with PTLD. **2D** Relation between the timing of remdesivir exposure and the subsequent development of the *de novo* RdRp V792I mutation in Case 2. A Ct value of 17.4 was obtained when the patient was readmitted with worsening pulmonary symptoms. A Ct value of 26 was obtained 32 days after the diagnosis of COVID-19 when the patient experienced marked improvement in symptoms and their oxygen requirement had resolved. **2E** Case 2 CT of the chest demonstrating multifocal nodules, many of which are surrounded by ground-glass opacities. Arrow indicates a cavitary lesion. An elevated galactomannan level from bronchoalveolar lavage fluid suggested the diagnosis of pulmonary aspergillosis.

24 days after the diagnosis of COVID-19, the patient was readmitted with worsening fatigue, cough, dyspnea, abdominal discomfort, and fever. Nasopharyngeal SARS-CoV-2 RT-PCR was again positive with a Ct of 24.4, and genomic sequencing identified Omicron BA.1.1. In the setting of a substantial oxygen requirement, the patient was treated with another five-day course of remdesivir and a ten-day course of dexamethasone. Genomic sequencing 38 days after COVID-19 diagnosis identified the *de novo* RdRp mutation V792I (G15814A) (Fig. 1). Figure 2A outlines the identification of this mutation in relation to prior remdesivir exposure.

In the setting of new abdominal swelling, computed tomography (CT) performed shortly after admission demonstrated a moderate right pleural effusion and mass-like soft tissue infiltration along the renal graft, contiguous with the abdominal wall (Fig. 2B). Further diagnostic evaluation yielded a serum Epstein Barr virus (EBV) viral load of 645,000 IU/mL as cytology from pleural fluid, flow cytometry from pleural fluid, and a retroperitoneal lymph node biopsy demonstrated cells suggesting EBV-positive diffuse large B-cell lymphoma (DLBCL) consistent with monomorphic post-transplant lymphoproliferative disorder (PTLD). The patient was treated with multiple cycles of anti-neoplastic therapy that included rituximab, cyclophosphamide, doxorubicin, vincristine, and prednisone. Three weeks after the initiation of chemotherapy, the patient developed new ulcerative, bullous, and necrotic cutaneous findings overlying the renal graft (Fig. 2C). Biopsy of these changes yielded EBV-positive lymphocytes consistent with PTLD. During the course of chemotherapy, the patient experienced severe cytopenias, gastrointestinal bleeding, and hemorrhagic shock. The patient progressed to ESRD, and belatacept and mycophenolate were discontinued. Encountering multiple other complications including cytomegalovirus viremia and bacterial infections over the course of an admission spanning three months, the patient’s cough, fever, and hypoxemia all resolved, their skin findings improved, EBV viremia decreased dramatically, and interval reimaging demonstrated decreased size of the patient’s renal graft and associated lymphadenopathy. Three months after the initial diagnosis of COVID-19, IgGs to SARS-CoV-2 nucleoprotein and spike were detectable, the Ct increased to 29.3, and the patient remained free of any symptoms suggesting active respiratory infection.

110 days after COVID-19 diagnosis, the patient developed a new onset of dry cough and rhinorrhea. Nasopharyngeal RT-PCR was positive for SARS-CoV-2 with a Ct of 23.3 (Omicron BA.1.1). RT-PCR for other respiratory pathogens was negative. Repeat RT-PCR one week later yielded a Ct of 22.6, and genomic sequencing at that time identified a *de novo* synonymous mutation in RdRp at K890 (Fig. 1). Providers monitored the patient’s mild symptoms, which gradually improved over the course of weeks. Ct 153 days after COVID-19 diagnosis performed when the patient was asymptomatic was 34.1. During the patient’s prolonged course of infection, two additional *de novo* non synonymous mutations were also identified in nsp6 and orf3 **(Supplemental Table 1).**

## Case 2

A patient in their 50s with a history of vascular disease, splenectomy, and diabetes underwent DDKT addressing ESRD. Prior to transplant, the patient received two doses of the mRNA-1273 vaccine. After receiving induction immunosuppression including methylprednisolone and ATG, the patient was maintained on prednisone, mycophenolate, and tacrolimus. DGF complicating the early postoperative period prompted the empiric administration of methylprednisolone. Graft biopsy performed one week after transplant demonstrated evidence of CNI toxicity, and the patient transitioned from tacrolimus to everolimus. Though graft function improved, the patient experienced a series of complications including medication-induced cytopenias, breakthrough cytomegalovirus viremia, and recurrent bacterial infections over the next several months.

Fourteen months after transplant, the patient developed malaise, shortness of breath, and cough. Nasopharyngeal PCR was positive for SARS-CoV-2, and in the setting of pulmonary infiltrates on x-ray and hypoxemia, the patient received a three-day course of remdesivir and a four-day course of baricitinib. SARS-CoV-2 genomic sequencing performed on day seven of illness identified a *de novo* V792I (G15814A) mutation in RdRp (Fig. 1). The patient’s symptoms improved significantly, hypoxemia resolved over the course of a few days, and the patient was discharged. 18 days after COVID-19 was diagnosed, the patient was readmitted with worsening cough and symptomatic hypoxemia. Nasopharyngeal RT-PCR detected SARS-CoV-2 with a Ct of 17.4. X-ray demonstrated worsening patchy infiltrates. Initially managed with methylprednisolone and a five-day course of remdesivir, the patient developed worsening hypoxemia requiring high-flow oxygen, and CT eventually identified multiple cavitary lung lesions (Fig. 2E). An elevated galactomannan level on bronchoalveolar lavage suggested the diagnosis of aspergillosis, and the patient’s symptoms and hypoxemia gradually resolved over the course of two weeks with the administration of voriconazole. Of note, *de novo* mutations in the nsp14 exonuclease (Fig. 1) and spike protein **(Supplemental Table 1)** were also identified during the course of infection.

## Discussion

As the COVID-19 pandemic has progressed, clinicians have increasingly recognized the role that immunosuppression plays in complicating the course of infection. Despite undergoing vaccination prior to transplant, the patient described in Case 1 experienced a protracted course of symptomatic infection that lingered over the course of several months. Similarly striking presentations have occurred in other patients treated with rituximab^6,7^. Compared to other immunosuppressing medications, rituximab appears to be associated with higher rates of adverse outcomes during the course of COVID-19. The increased risk of mortality attributed to rituximab extends to patients treated for rheumatologic and oncologic indications^8^. Like Case 1, the previously vaccinated SOT recipient described in Case 2 also experienced life-threatening complications related to COVID-19 including severe pulmonary aspergillosis. Sequencing and RT-PCR analysis in both cases strongly suggested the presence of persistent, viable SARS-CoV-2 infection contributing to ongoing symptoms.

When ineffective immune clearance contributes to persistent viral replication in immunocompromised hosts, an increased opportunity for mutation arises. The SARS-CoV-2 non-structural protein, nsp12, encodes the most rapidly replicating RdRp of any known virus. While the exonuclease nsp14 and its co-factor nsp10 offset the low fidelity of nsp12, the frequency at which SARS-CoV-2 generates mutations remains substantial^9,10^. In the setting of remdesivir exposure and profound immune deficits permitting unchecked replication over an extended period of time, virus isolated from both of the patients in this report independently developed a *de novo* V792I substitution in ORF1ab, the open reading frame encoding nsp12. A recent study showed that V792I readily develops *in vitro* after serial cell passage conducted in the presence of increasing remdesivir concentrations^5^. Out of the 11 million genomes deposited in GISAID globally, fewer than 300 isolates contain the V792I substitution^11^. Interestingly, V792I was identified in roughly 7% of genomes observed in a recent pre-publication series of immunocompromised patients^12^. Whereas remdesivir incorporated into the elongating RNA of wild-type virus inhibits the integration of a complementary uridine-triphosphate nucleoside, V792I effectively permits a lower uridine-triphosphate concentration to overcome the inhibitory effect of remdesivir. This substitution alone has been demonstrated to increase the remdesivir half-maximal effective concentration (EC50) by 2.6-fold^5^. Other mutations observed *in vitro* may complement the effect of V792I via distinct mechanisms to further increase the remdesivir EC50^5^. Notably, SARS-CoV-2 isolated throughout the course of infection in both Case 1 and 2 also exhibited the P323L substitution, an Omicron-defining mutation, in ORF1ab that has been associated with a modest increase in the remdesivir EC50^13^. The combined effect of these mutations and others may limit the clinical efficacy of remdesivir.

Our work emphasizes the potential risk of immune escape in immunocompromised hosts, a scenario in which novel spike mutations evade a suboptimal immune response and contribute to recrudescence of symptomatic infection. Equally concerning are recent reports in immunocompromised hosts highlighting the proclivity of SARS-CoV-2 to develop spike mutations conferring resistance to immunotherapeutics after treatment with monoclonal antibodies^14^.

As remdesivir use has become widespread, and we show that mutations associated with remdesivir resistance arise *in vivo*, our work emphasizes the importance of augmented surveillance efforts to detect clinically significant mutations in immunocompromised patients. Potentially foreshadowing an “endgame” scenario for the COVID-19 pandemic, complex cases like the ones described in this report may presage the eventual need for more advanced molecular diagnostics at the onset of illness to guide therapeutic decisions. It is reasonable to assess for new mutations in patients with prolonged illness who experience persistent infection despite initial therapy.

Finally, the prolonged nature of the infections outlined in this work emphasizes the critical need for thoughtful input from specialists when considering isolation precautions in immunocompromised hosts. Despite a Ct value approaching 30, detectable anti-SARS-CoV-2 IgG, and markedly improved symptoms three months after COVID-19 diagnosis, the patient described in Case 1 eventually experienced relapsed symptomatic infection associated with a decreasing Ct and newly detected mutations in spike and ORF1ab. Multiple authors have posited that several variants of concern may have originated in immunocompromised hosts^15,16^. The failure to appropriately identify, treat, and control the spread of mutated SARS-CoV-2 isolates could have far-reaching consequences.

## Methods

### SARS-CoV-2 sequencing and bioinformatic analysis.

Viral genome sequencing was carried out as described previously^17^ using the XGen SARS-CoV-2 amplicon-based library prep method (IDT, Coralville, Iowa). Sequencing was performed on an Illumina NovaSeq 6000, 150PE, dual index run. Sequencing reads were demultiplexed using the Illumina bcl2fastq2 Conversion Software v2.20 and adapters and low-quality bases were trimmed with Trimmomatic v0.36. BWA v0.7.17 was utilized for mapping reads to the SARS-CoV-2 reference genome (NC_045512.2, wuhCor1). Panel-specific tiled primer sequences were removed using Primerclip v.0.3.8. BCFtools v1.9 was used to call mutations and assemble consensus sequences. Phylogenetic lineage was assigned using Pango nomenclature^18,19^ (v.4.0.6 PLEARN-v1.8). Nextclade v2.0.0 and Auspice v2.37.3 phylogenomic visualization Nextstrain project) were used to examine viral genome clade assignment and mutation calling.

#### Structural analysis

3D structures were created with UCSF ChimeraX 1.4^20^. Structural overlay was done using MatchMaker in ChimeraX. Chain pairing was performed according to the best aligning pairs of chains between reference and match structure.
